# A Study of *Ser217Leu* and *Ala541Thr* Polymorphism in the Men Afflicted with Prostate Cancer and in the Men being Suspicious of Prostate Cancer

**DOI:** 10.31557/APJCP.2020.21.6.1551

**Published:** 2020-06

**Authors:** Zahra Zahiri, Fatemeh Zahiri

**Affiliations:** *Department of Genetics scholar Biology sciences Islamic Azad university of Tonekabon Branch, Tonekabon, Iran. *

**Keywords:** Ser217Leu, Ala541Thr, polymorphism, prostate cancer

## Abstract

**Background and objective:**

Prostate cancer is one of the most widespread cancers among men throughout the world. In addition, it is the second cause of death after lung cancer. Occurrence of the prostate cancer is variable in various regions of the world. Solely, there are three known risk factors for the prostate cancer, including: Age, inheritance and ethnic origin. ELAC2 protein is a phosphodiesterase enzyme encoded by *ELAC2* gene in human. This gene is placed on chromosome 17, and it is believed that product of the mentioned gene is an endonuclease contributed in puberty of mitochondrion’s tRNA. From clinical viewpoint, variables of *ELAC2* gene such as *Ser217Leu* and *Ala541Thr* Missense mutations which are accompanied by hereditary prostate cancer (*HPC2*).Objective of this study is to investigate *Ser217Leu (rs4792311)* and *Ala541Thr (rs5030739) *polymorphisms in the individuals with prostate cancer or those who are suspicious of prostate cancer with family past record/history.

**Study method:**

In this study conducted by case-control method in 2018, 102 men with prostate cancer and 98 men being suspicious of prostate cancer out of 10 families referred to shahid Rajaei Hospital in Tonekabon county to study and check were investigated. After collection of data using questionnaire, sampling from individuals and performance of the rest steps, study of polymorphism was carried out by PCR sequencing technique, and the results were analyzed by Chromas software.

**Finding:**

Of the total studied 102 individuals, 44 individuals (43.1%) were homozygote for *Ser217Leu* mutation, 36 individuals (35.2%) were heterozygote and 22 individuals (21.5%) lacked Missense mutation. for Ala541Thr mutation, 18 ones (17.6%) were heterozygote and 84 ones (82.3%) lacked Missense mutation. For *Ser217Leu* mutation, out of 98 suspicious individuals, 21 individuals (21.4%) were homozygote. 6 individuals (6.1%) were heterozygote and 71 individuals (72.4%) lacked the mutation. For Ala541Thr mutation, 15 ones (15.3%) were homozygote and 84 ones (84.6%) lacked the studied mutation.

**Conclusion:**

Results of this research showed that, in the individuals with the prostate cancer, there is a relationship between *Ser217Leu *and *Ala541Thr* polymorphism of *ELAC2* gene and/with prostate cancer, and the suspicious individuals gotten involved in the mutation must take action to prevent this cancer.

## Introduction

Prostate (which means protector) is a tubule alveolar exocrine gland of reproduction system in men. Among the different species, prostate is different from anatomical, chemical and physiological viewpoint noticeably. 

Operation of the prostate is in such a way that it secretes an alkaline, milky or white-colored liquid which, almost, constitutes 30% of sperm volume along with sperm and semen liquid in human. Generally, sperm is to be alkalinized by secretions of the other glands, including minimum liquid rate of the semen liquid. Alkalinity of the semen liquid leads to neutralization of acidity in the vagina system and increases longevity of sperm. Also, prostate contains some smooth muscles which assists in discharge of sperm during ejaculation (Hugginset al., 1942). Classic description of a healthy man’s prostate appears that it is a little bigger than a walnut. Average weight of natural prostate in adult men is 11 grams approximately which is ranged from 7 to 16 grams (Leissner and Tisell, 1979).

Secretions of the prostate are different among the various species. Generally they are consisted of simple sugars and often a little alkaline. In secretions of the human prostate, ingredient of protein is less than 1% and includes proteolytic enzymes, , Beta micro semeno protein and specific antigen of prostate (Ali and Ahmed, 2017)

Approximately, 20,000 Protein-encoding genes have been expressed in the human cells, and almost, 75% of these genes are expressed in natural prostate. Roughly, 150 cases of these genes are expressed in prostate specifically, and 20 genes have a high specificity for prostate. The related specific proteins are expressed in the glandular and secretory cells of prostate and have some actions being important for specificity of semen. Some samples of the most specific proteins of prostate are it’s specific enzymes, including prostate specific antigen (PSA) and ACPP protein (Ali and Ahmed, 2017).

Prostatitis is an inflammation of the prostate gland. Basically, there are four types of different prostatitis.each of which is accompanied by various causes and results. It’s two relatively uncommon forms are as follows: Acute prostatitis and chronic bacterial prostatitis which are treated by 1 and 2 – rank antibiotics. Non–bacterial chronic prostatitis or pelvic chronic pain syndrome (The third rank) which includes 95% of prostatitis can be treated by a variety of treatment methods, including Alpha blockers, physiotherapy, psychotherapy, antihistamines, anxiolytics, modulators of nerve, surgery, and…etc. The fourth prostatitis group is uncommon in the public population relatively and counted as a sort of leukocytosis (Christensen and Andriole Jr, 2009).

Only, there are three known risk factors for the prostate cancer: Age, inheritance and ethnic origin. Its outbreak increases after sixtieth decade of life intensively. Yet, autopsy studies showed that risk of affliction with prostate cancer from age of 30 to higher than 30 is related to the risk factor depended on age. (Soos et al., 1993; Sakr et al., 2005). A few other risk-maker factors regarding the prostate cancer have been studied. Overweight and obesity as the suggested risk-maker factors have been studied, but no potential relationship of them with the prostate cancer has been found (Bianchini et al., 2002).

High consumption of fat has been studied in the food regimen, and a weak relationship may have been existed with risk of affliction by the prostate cancer due to its high consumption (Dagnelie et al., 2004). Prostate specific antigen (PSA) is a glycoprotein produced in epithelium of prostate glands. It’s known performance is liquid making of semen after ejaculation. Blood level of prostate specific antigen (PSA) suggests relationship with risk of affliction with prostate cancer, but it doesn’t suggest specificity for the prostate cancer at all (Stamey et al., 1987).

For various reasons, including increase of benign hyperplasia of prostate, infection of urethras / urinary ducts and prostatitis, this level can be increased (Stamey et al., 1987).

It has been specified that the prostate cancer is in cluster form within some families. Analysis shows that this family clustering forms can be described by, at least, a dominant rare gene of sensitivity. On the basis of relational studies, five cancerous sensitive loci have been assumed for the prostate cancer, especially for inherited prostate cancer: Those loci whose HPC1 are restricted to *q24-251 *chromosome, those loci whose PCAP are limited to *q42.2-431*, those loci whose CAPB are restricted to P361, those loci whose HPCX are limited to *Xq27-28* and those loci whose *HPC20* are restricted to q1320. Topical simulation and screening of the mutations introduced a gene named *HPC2*/*ELAC2* as suppressor of prostate cancer which this gene possesses the hazardous mutations related to relational prostate cancer (Wang et al., 2001) *ELAC2* protein is an enzyme on phosphodiesterase encoded by *ELAC2* gene in human (Rebbeck et al., 2000; Noda et al., 2006).

*ELAC2* gene encodes a 92-kilo-Dalton protein and is connected to nucleus. *ELAC2* is a zinc phosphodiesterase showing activity of tRNA3 endonuclease within mitochondrion. Mitochondrion contains a reservoir of tRNAs interfering in transfer of subunit-13 protein of respiratory chain encoded by mitochondrion’s genome. In puberty of tRNA, *ELAC2* acts through omission of additional –3^’ (Three additional nucleotides) from tRNA precursors and produces t^’RNA3 end (Terminus). This reaction leaves behind a 3’–hydroxyl group at the end of tRNA and places a 5^’–phosphoril group in another cut end (Terminus). This reaction requires zinc ions as cofactors of reaction (Brzezniak et al., 2017) From clinical viewpoint, variables of the *ELAC2* gene are related to hereditary prostate cancer. (*HPC2*)2. Several mutations, including shortening and Missense mutations as cause of this disease are counted to be based on analysis of topical bond and simulation. One of the cases of important Missense mutation which occurs in *ELAC2* can be referred to conversion of Serine amino acid into Leucine and, also, Alanine into Threonine. In addition, mutation in *ELAC2* leads to shortage of oxidative phosphorylation [COXPD17] 17 which is a recessive autosomal rare disorder of mitochondrial operation established by intensive hypertrophic cardiomyopathy (Wang et al., 2001; Haack et al., 2013).

Fogiwara et al., (2002) genotyped *S217L* and *A541T* variants within a Japanese group, include 350 patients with prostate cancer, 242 man/male populations as control and 114 exposed-to-low danger individuals. Both two *Leu217* and *Thr541* alleles were higher in the patients than control group, and probable ratio along with these variants was higher in Japan than Caucasia. *Leu217* and *thr541* polymorphisms were widespread in Japan less than Caucasia, but both two polymorphisms established increasing risk of affliction with prostate cancer in Japan noticeably (Fujiwara et al., 2002).

Tavtigian et al., (2001) embarked on an accurate mapping in a set bigger than 33 families using condensed markers in P1117 region. Analysis of sequence of *HPC2*/*ELAC2* gene showed four types of sequence, including rare changes of shift frame and three Missense changes. Two types of these variants were accompanied by the prostate cancer. Change of Serine into Leucine in 217 amino acid (*Ser217Leu*) and change of Alanine into Threonine in 541 amino acid. (*Ala541Thr*) Frequency of *Leu217* allele among the cases of HPC compared to the men married the studied races without being under infleunce was higher. Also, *Thr541* allele in the HPC cases was more widespread than control group.

Rebbek et al., (2002) showed that risk of affliction with the prostate cancer (*HPC2*) was increased in the individuals who carry one or both two prevalent Missense variants identified by Tavtigian et al. These Missenses included mutation of *Leu217* allele from *Ser217* polymorphism to leu *(S217L) *polymorphism and mutation of *Thr541* allele from *ala541* polymorphism to (*A541T*) Thr polymorphism. *A541T* has been placed next to the protected Motif histidine suggesting that presence of thr541 allele may have harmful effects on performance of the protein encoded by this gene (Rebbek et al., 2009).

*Study method*

In this study conducted by case-control method in 2018, 102 men with prostate cancer and 98 men being suspicious of prostate cancer out of 10 families referred to Shahid Rajaei Hospital in Tonekabon county were studied. Criteria for entrance into the study included as following: Availability/possession of an individual with the prostate cancer in family such as grandfather, father, brother, uncle, aunt and …etc, lack of affliction with medical diseases (Cardiac, pulmonary and renal diseases and increase of blood pressure/hypertension), lack of addiction to cigarette or another special substance and satisfaction with participation in the research. In this study, sampling was conducted in available sampling form. After some descriptions concerning how to research and its objectives for the individuals participated in the research and acquirement of their satisfaction, the interview form, including individual information such as age of individuals, relationship with individuals afflicted by the prostate cancer and …etc was completed. Check list of the patients’ investigation following the study of book and articles was regulated and confirmed by a few university professors. Genomic DNA was extracted from environmental blood leukocytes by use of BIO BASIC kit. For the purpose of qualitative study of appropriate DNA extraction, the samples were electrophoresized on the 1% Agarose gel and, for the purpose of quantitative study of purity in the extracted DNA, 260 to 280 proportion was used. Following DNA extraction of the samples, specific primers were designed by use of Generunner software, and blast was carried out in NCBI site through online form. Sequence of primers used in this research have been presented in table No1. 

Then, duplication of the considered fragments was conducted using the primers with the aid of PCR technique. PCR plan, in initial denaturation form, was performed in 95^o^ for five minutes; then, 35 cycles of denaturation were enforced in 95^o^C for 30 seconds, elongation was carried out in 72^o^C for 30 seconds and, at the end, final elongation was fulfilled in 72^o^C for 5 minutes. Later, in order to make sure of successful duplication of the considered fragment, 10μl of PCR product was tested on the 1.5% Agarose gel and, for the purpose of more accurate investigation, PCR product was sent for sequencing to the domestic company of ‘‘Gene-Technologists’’. And, results of sequencing were analyzed by chromas software.

## Results

Out of 102 men with the prostate cancer for the purpose of investigation into *Ser217Leu(rs4792311)*, 44 ones (43.1%) were homozygote and Missense mutation existed in them, and Serine amino acid was converted into Leucine within them; 36 ones (35.2%) were heterozygote and Missense mutation existed in them, and Serine amino acid was converted into leucine within them. 22 individuals (21.5%) of them laced *Ser217Leu* mutation. Out of 102 individuals for the purpose of *Ala541Thr (rs5030739)*, 18 individuals (17.6%) were heterozygote and Missense mutation existed in them, and Alanine amino acid was converted into Threonine within them. 84 individuals of them (82.3%) lacked this mutation. No any heterozygous sample was observed in them. From amongst these studied 102 individuals, 8 individuals (7.8%) had both two *Ser217Leu* and* Ala541Thr* mutations. Out of 98 suspicious individuals, 21 individuals (21.4%) were homozygote in order to study* ser217leu* mutation. 6 individuals (6.1%) was heterozygote and 71 individuals (72.4%) lacked mutation.

For *Ala541Thr* mutation, 15 individuals (15.3%) were homozygote and 84 individuals (84.6%) lacked the studied mutation.

**Figure 1:**
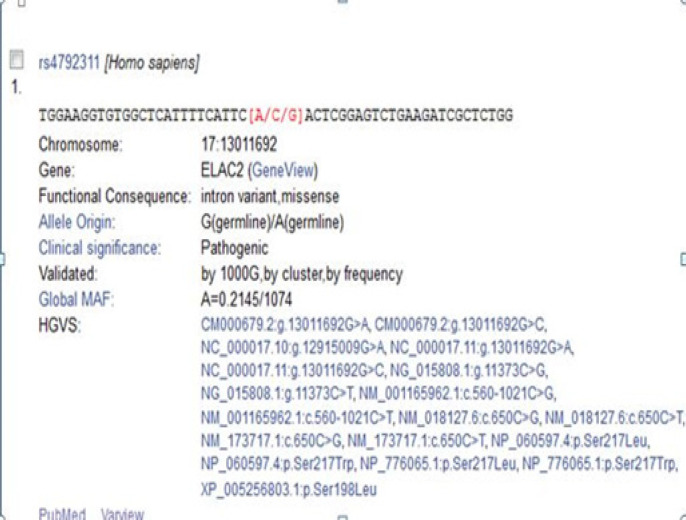
The RSSNP search for rs5030739 Mutation of *ELAC2* Gene

Figure 1 shows the RSSNP search for rs4792311 and rs5030739 mutation of *ELAC2* gene. 

**Figure 2:**
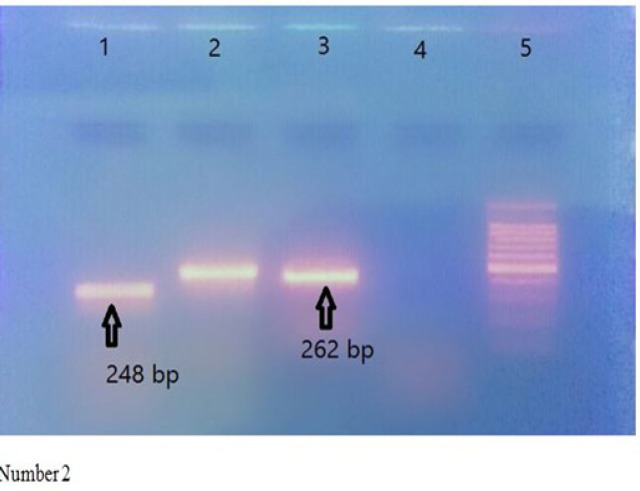
Observance of Electrophoresis's Result in PCR Product. Sump/small well No1 suggests Ala541Thr mutation with a 248-base-pair band, sump No2 and 3 suggests ser217Leu mutation with a 262-base-pair band, sump No4 is negative control and sump No5 is lader

Figure 2 shows observance of electrophoresis’s result in PCR product. Sump/small well No1 suggests *Ala541Thr* mutation with a 248-base-pair band, sump No2 and 3 suggests *ser217Leu *mutation with a 262-base-pair band, sump No4 is negative control and sump No5 is lader. 

**Figure 3:**
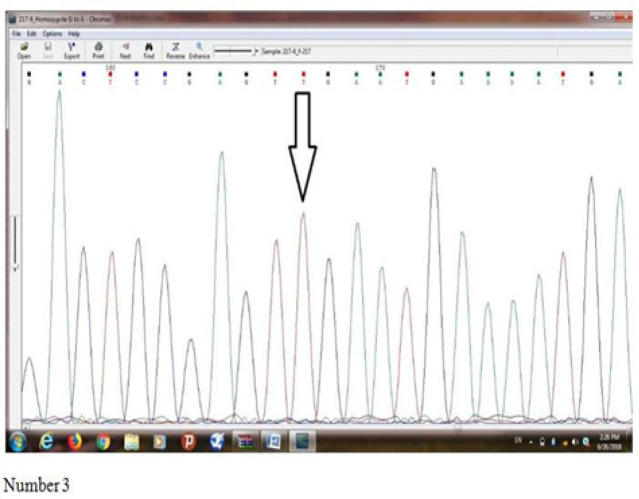
Indicates Results of Sequence Determination of Ser217 Leu (rs4792311) Mutation of *ELAC2* Gene in an Individual with Prostate Cancer which a Homozygote Mutation was Observed in him/her. Base T represents Missense mutation, and Serine amino acid has been converted into Leucine amino acid

Figure 3 indicates results of sequence determination of S*er217 Leu (rs4792311)* mutation of *ELAC2* gene in an individual with prostate cancer which a homozygote mutation was observed in him/her. Base T represents Missense mutation, and Serine amino acid has been converted into Leucine amino acid.

**Figure 4:**
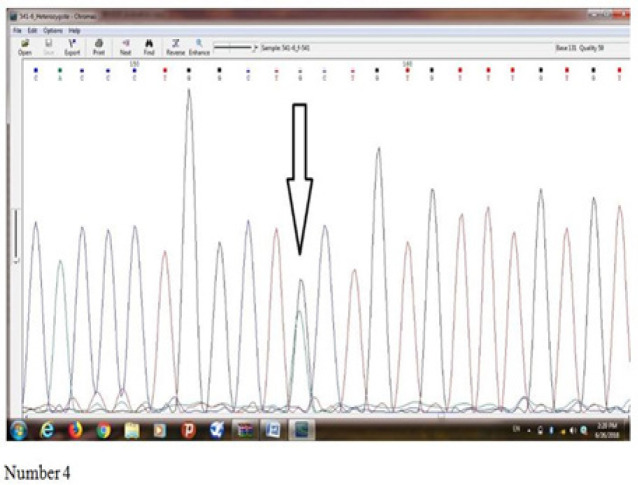
Reveals Results of Sequence Determination of Ala541Thr (rs5030739) Mutation of ELAC2 Gene in an Individual with Prostate Cancer which a Heterozygote Mutation was Observed in him/her. Base G suggest Missense mutation, and alanine amino acid has been converted into threonine amino acid

Figure 4 reveals results of sequence determination of *Ala541Thr (rs5030739)* mutation of *ELAC2* gene in an individual with prostate cancer which a heterozygote mutation was observed in him/her. Base G suggest Missense mutation, and alanine amino acid has been converted into threonine amino acid.

**Figure 5:**
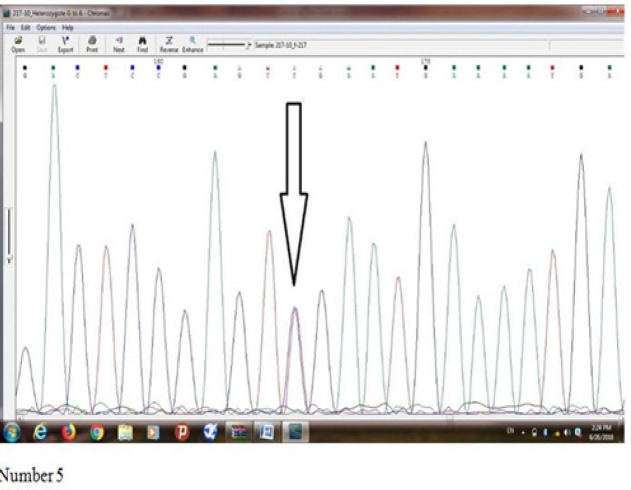
Shows Results of Sequence Determination of Ser217Leu (rs4792311) Mutation of ELAC2 Gene in an Individual with Prostate Cancer which a Heterozygote Mutation was Observed in him/her. Base T represents Missense mutation, Serine amino acid has been converted into Leucine amino acid

Figure 5 shows results of sequence determination of *Ser217Leu (rs4792311)* mutation of *ELAC2* gene in an individual with prostate cancer which a heterozygote mutation was observed in him/her. Base T represents Missense mutation, Serine amino acid has been converted into Leucine amino acid.

**Figure 6:**
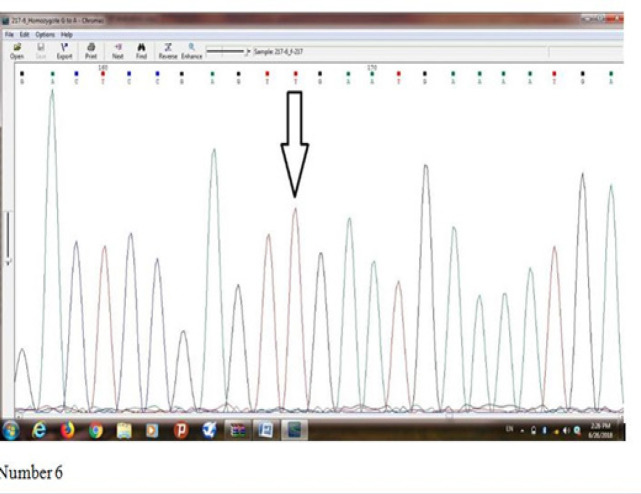
Indicates Results of Sequence Determination of Ser217Leu (rs4792311) Mutation of ELAC2 Gene in an Individual who is Suspicious of Prostate Cancer which Homozygote Mutation was Observed in him/her. Base T represents Missense mutation, and Serine amino acid has been turned into Leucine amino acid

Figure 6 indicates results of sequence determination of *Ser217Leu(rs4792311)* mutation of *ELAC2* gene in an individual who is suspicious of prostate cancer which homozygote mutation was observed in him/her. Base T represents Missense mutation, and Serine amino acid has been turned into Leucine amino acid.

**Figure 7.**
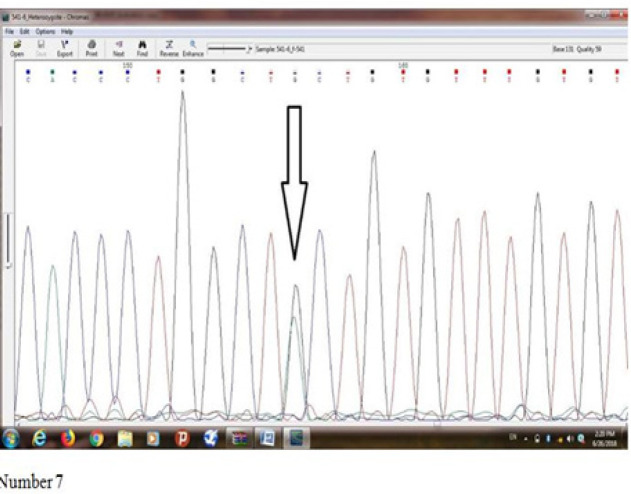
Shows Results of Sequence Determination of Ala541Thr (rs5030739) Mutation of ELAC2 Gene in an Individual being Suspicious of Prostate Cancer which Heterozygote Mutation was Observed in him/her. Base G suggests Missense mutation, and Alanine amino acid has been converted into Threonine amino acid

Figure 7 shows results of sequence determination of *Ala541Thr (rs5030739)* mutation of *ELAC2* gene in an individual being suspicious of prostate cancer which heterozygote mutation was observed in him/her. Base G suggests Missense mutation, and Alanine amino acid has been converted into Threonine amino acid.

Table 2 indicates the results achieved from NGS for each one of the studied mutations.

**Table 1:** Information on The Primers *Ser217Leu* and Primers *Ala541Thr* Used in This Study

Ser217Leu primers			
primers	Sequence	Tm	PCR product
Forward	GCTGATTTAATTGGCGTTCTGGC	63.5	262bp
Reverse	CAAGCCTTTCTGCTGCTCTGT	63.5	262bp
Ala541Thr primers			
Primers	Sequence	Tm	PCR product
Forward	TCCAAAGCAGACATCAGCCTC	63	248bp
Reverse	GCTCCAGCTTTGTGGTCCAG	63.5	248bp

**Table 2:** The Results Achieved from NGS for Each One of the Studied Mutations

Mutation	Chromosome	position	REF	ALT	quallity	Genotype	effect	Gene	Type of translation	RS	Disease
Ser217Leu	17	12.915.009	G	A	3393.77	homozygous	Missense	*ELAC2*	Coding	rs4792311	Prostat canser
Ala541Thr	17	12.899.902	C	T	538.77	homozygous	Missense	*ELAC2*	Coding	rs5030739	Prostat canser

## Discussion

Prostate cancer is one of the most widespread cancers among men throughout the world. Rate of occurrence of the prostate cancer is very different among the various regions of the world. The highest rate of the occurrence was observed in the Northern America and the least rate in the Southeast of the Asia. It has been estimated that 1.1 millions of men with the prostate cancer were diagnosed in 2012 out of which 70% (795,000 cases) have been in the developed countries. (Jemal et al., 2010; Stewart and Wild, 2017) Only, there are three known risk maker factors for the prostate cancer: Age, inheritance and ethnic origin. Death risk as a result of this disease is increased in case of diagnosis in the young people. Also, it has been showed that the men who are in families facing with a hereditary form of the prostate cancer caused by *BRCA2* mutation increase risk of affliction with prostate cancer (Soos et al., 2005). *ELAC2* protein is a phosphodiesterase zinc enzyme encoded by *ELAC2* gene in the human. This gene is placed on the chromosome 17, and it is imagined that product of this gene is an endonuclease contributed in puberty of mitochondrial tRNA (Noda et al., 2006). Variables of *ELAC2* gene are accompanied by hereditary prostate cancer. (*HPC2*)2 One of important cases of Missense mutation occurred in *ELAC2* gen, it can be referred to conversion of Serine into Leucine and, also, conversion of Alanine into Threonine (Haack et al., 2013).

In this study, 102 men with prostate cancer and 98 men being suspicious of prostate cancer out of 10 families referred to the TONEABON Shahid Rajaei hospital to study and check were investigated. Out of 102 men with the prostate cancer, 44 ones (43.1%) were homozygote for the purpose of studying the *Ser217Leu (rs4792311)* and Missense mutation existed in them, and Serine amino acid was converted into Leucine amino acid within them. 36 individuals (35.2%) were heterozygote and Missense mutation existed in them, and Serine amino acid was turned into Leucine amino acid within them.

22 individuals out of them lacked *Ser217Leu *mutation. For investigation into *Ala541Thr (rs5030739) *mutation, 18 individuals (17.6%) out of 102 individuals were heterozygote and Missense mutation existed in them, and Alanine amino acid was converted into Threonine amino acid within them. 84 individuals out of them (82.3%) lacked this gene. No any homozygote sample was observed in them. Out of these studied 102 individuals, 8 individuals (7.8%) had both two *Ser217Leu* and *Ala541Thr* mutations. From amongst 98 suspicious individuals, 21 individuals (21.4%) were homozygote for investigation into* Ser217Leu* mutation. 6 ones (6.1%) were heterozygote and 71 ones (72.4%) lacked the mutation. For Ala541Thr mutation, 15 individuals (15.3%) were homozygote and 84 individuals (84.6%) lacked the studied gene. Camp and Tavtigation, (2002) carried out a meta-analysis for the data related to relationship of* Ser217Leu* and *Ala541Thr* alleles in the *ELAC2* (*HPC2*) gene and prostate cancer. They observed that two variants of *Ser217Leu* and *Ala541Thr *mussense mutation were related to family prostate cancer. In another study in 2003, Takashi et al embarked on investigation into a relationship of* ser217leu *polymorphism of *HPC2*/*ELAC2* gene with the men being suspicious of the prostate cancer in Japan. These Japanese researchers concluded that *HPC2*/*ELAC2* somatic mutations in the prostate cancer are uncommon. In this manner, they realized that imbalance of allele in genic locus and changes in gene expression are rare. Although a difference was not reported in frequency of *ser217leu *allele among the patients with the prostate cancer and control within the country’s western population, they showed that this polymorphism is a potential index of risk of affliction with prostate cancer in the Japanese men and must be studied in other ethnic groups (Takahashi et al., 2003).

Xu et al., (2010) performed a metaanalysis from 18 studies which concerned with studying of a relationship between *Ser217leu *and *Ala541Thr* polymorphism of *ELAC2* gene and risk of affliction with the prostate cancer. In the analysis classified for *Ser217leu* polymorphism, risk of affliction with prostate cancer in the Asian and Caucasian population and the studies used in the cases of dispersed and family prostate cancer increased at noticeable rate. In the analyses classified for *Ala541Thr *polymorphism, similar results were found in the Asian population. This metaanalysis showed that *Ser217Leu* and *Ala541Thr *polymorphism of *ELAC2* gene is accompanied by increase of risk of prostate cancer and may be the markers which are sensitive to low penetration of the prostate cancer (Meitz et al., 2002). 

In his study, Dong showed that two prevalent Missense variants of* Ser217leu* and *Ala541Thr* (mutation) of *HPC2*/*ELAC2* gene are not able to change enzymatic activities of *ELAC2* and, also, indicated that these two variants are related to the prostate cancer in the samples of the men whose families are afflicted with hereditary prostate. Yet, in analysis of not-selected cases and control for family history of the prostate cancer which majority of them had prostate cancer, only carries of both two *Leu217* and *Thr541* alleles increased risk of affliction with prostate cancer, and risk of affliction with cancer a significant relationship with their family short history or race (Dong, 2006).

Other studies and present study suggest importance of the studied polymorphisms in process of affliction with the prostate cancer. Results of the current research showed that, in 102 individuals who were afflicted with prostate cancer and not selected for family history, there exists a significant relationship between *Ser217leu (rs4792311) *and *Ala541Thr (rs5030739)* polymorphisms, and Missense mutation established in these individuals was accompanied by risk of affliction with the prostate cancer. Out of these 102 individuals, 8 individuals (7.8%) had both two Ser217leu and Ala541Thr mutations suggesting both two variants of *Ala541thr* and *Ser217leu* widespread Missense increase risk of affliction with prostate cancer in individuals. Of 98 individuals who were suspicious of the prostate cancer and selected for family history, both two studied mutations were observed in some individuals and rate of *Ser217leu* mutation compared to *Ala541Thr *was higher in these individuals, and these individuals may be exposed to risk of affliction with the prostate cancer and must take the required measures for prevention. Possibly, there exists a significant relationship between the studied mutations and hereditary prostate cancer, and individuals who are in a family with history of the prostate cancer must be investigated in lower ages. Generally, in 200 individuals studied in this research, rate of *Ser217leu *mutation has been higher than that of *Ala541Thr *mutation, and relationship between *Ser217Leu* variant and the prostate cancer is stronger.
